# Improving Walking Economy With an Ankle Exoskeleton Prior to Human-in-the-Loop Optimization

**DOI:** 10.3389/fnbot.2021.797147

**Published:** 2022-01-10

**Authors:** Wei Wang, Jianyu Chen, Jianquan Ding, Juanjuan Zhang, Jingtai Liu

**Affiliations:** ^1^College of Artificial Intelligence, Institute of Robotics and Automatic Information System, Nankai University, Tianjin, China; ^2^Tianjin Key Laboratory of Intelligent Robotics, Nankai University, Tianjin, China

**Keywords:** wearable device, ankle exoskeleton, walking assistance pattern, biomechanics, human-in-the-loop optimization, walking economy

## Abstract

Lower limb robotic exoskeletons have shown the capability to enhance human locomotion for healthy individuals or to assist motion rehabilitation and daily activities for patients. Recent advances in human-in-the-loop optimization that allowed for assistance customization have demonstrated great potential for performance improvement of exoskeletons. In the optimization process, subjects need to experience multiple types of assistance patterns, thus, leading to a long evaluation time. Besides, some patterns may be uncomfortable for the wearers, thereby resulting in unpleasant optimization experiences and inaccurate outcomes. In this study, we investigated the effectiveness of a series of ankle exoskeleton assistance patterns on improving walking economy prior to optimization. We conducted experiments to systematically evaluate the wearers' biomechanical and physiological responses to different assistance patterns on a lightweight cable-driven ankle exoskeleton during walking. We designed nine patterns in the optimization parameters range which varied peak torque magnitude and peak torque timing independently. Results showed that metabolic cost of walking was reduced by 17.1 ± 7.6% under one assistance pattern. Meanwhile, soleus (SOL) muscle activity was reduced by 40.9 ± 19.8% with that pattern. Exoskeleton assistance changed maximum ankle dorsiflexion and plantarflexion angle and reduced biological ankle moment. Assistance pattern with 48% peak torque timing and 0.75 *N*·*m*·*kg*^−1^ peak torque magnitude was effective in improving walking economy and can be selected as an initial pattern in the optimization procedure. Our results provided a preliminary understanding of how humans respond to different assistances and can be used to guide the initial assistance pattern selection in the optimization.

## 1. Introduction

Advanced lower limb exoskeletons were proposed as a kind of potential device to improve walking efficiency for healthy individuals or to assist rehabilitation and daily activities for patients with motor dysfunctions (Ferris and Young, 2017; Sawicki et al., 2020; Siviy et al., 2020). In recent years, ankle and hip exoskeletons have been used to reduce metabolic costs, during walking and running. For example, a tethered soft exosuit for assisting stroke recovery patients increased the wearer's paretic limb propulsion force and ankle dorsiflexion angle during treadmill and overground walking (Awad et al., 2017), and its portable version assisted both walking and running for able-bodied persons was able to reduce metabolic costs during these activities (Kim et al., 2019). One lightweight untethered ankle exoskeleton for assisting cerebral palsy individuals was capable of reducing the metabolic cost of transport and soleus (SOL) muscle activity and increasing over-ground walking speed (Orekhov et al., 2020). An unpowered passive-elastic knee-ankle exoskeleton that stores energy from knee extension and releases energy to assist ankle plantarflexion was able to reduce the metabolic power of walking (Etenzi et al., 2020).

Although exoskeletons have shown the ability to effectively enhance human locomotion, the pattern of the assistance can significantly affect device performance and wearing comfortability (Lee et al., 2017; Quinlivan et al., 2017; Nuckols et al., 2021). Both have been substantially improved by human-in-the-loop optimization (HILO) which continuously updates assistance patterns to generate customized assistance. Zhang et al. used an evolution strategy to optimize assistance patterns for an hour and the resulting pattern reduced metabolic energy cost by 24.2 ± 7.4% (Zhang et al., 2017b). Ding et al. used a Bayesian algorithm to identify hip extension assistance within 20 min and the optimized pattern was able to reduce metabolic cost by 17.4 ± 3.2% (Ding et al., 2018). During the optimization process, wearers usually experience a fairly large number of assistance patterns. For example, in Zhang et al.'s study (Zhang et al., 2017b), this number was 32. Under different assistance patterns, wearers may have different physiological and biomechanical responses. Some of these patterns may be uncomfortable for the wearers and lead to unpleasant optimization experiences. In addition, there is no knowledge of which assistance pattern is favorable to work as the initial one for HILO.

Therefore, we aimed to investigate the effectiveness of different assistance patterns on improving the walking economy on a lightweight cable-driven ankle exoskeleton. Considering that ankle joint is the largest contributor of forward propulsion during walking (Farris and Sawicki, 2012), we first built a tethered exoskeleton that provided ankle plantarflexion assistance during human walking. Assistive torque profile was produced by emulating biological ankle joint moment during level walking. Then we designed a series of assistance patterns within the parameter space of HILO. We measured and analyzed participants' metabolic costs, muscle activities, kinematics, and kinetics while walking with these assistance patterns. We expected our study to provide a preliminary understanding of how these assistance patterns influenced wearers' physiological and biomechanical characteristics during walking. Furthermore, the results of our study can help to improve the time efficiency and to reduce the discomfort of the ankle exoskeleton assistance pattern optimization process.

## 2. Methods

### 2.1. System Overview

**Figure 1:**
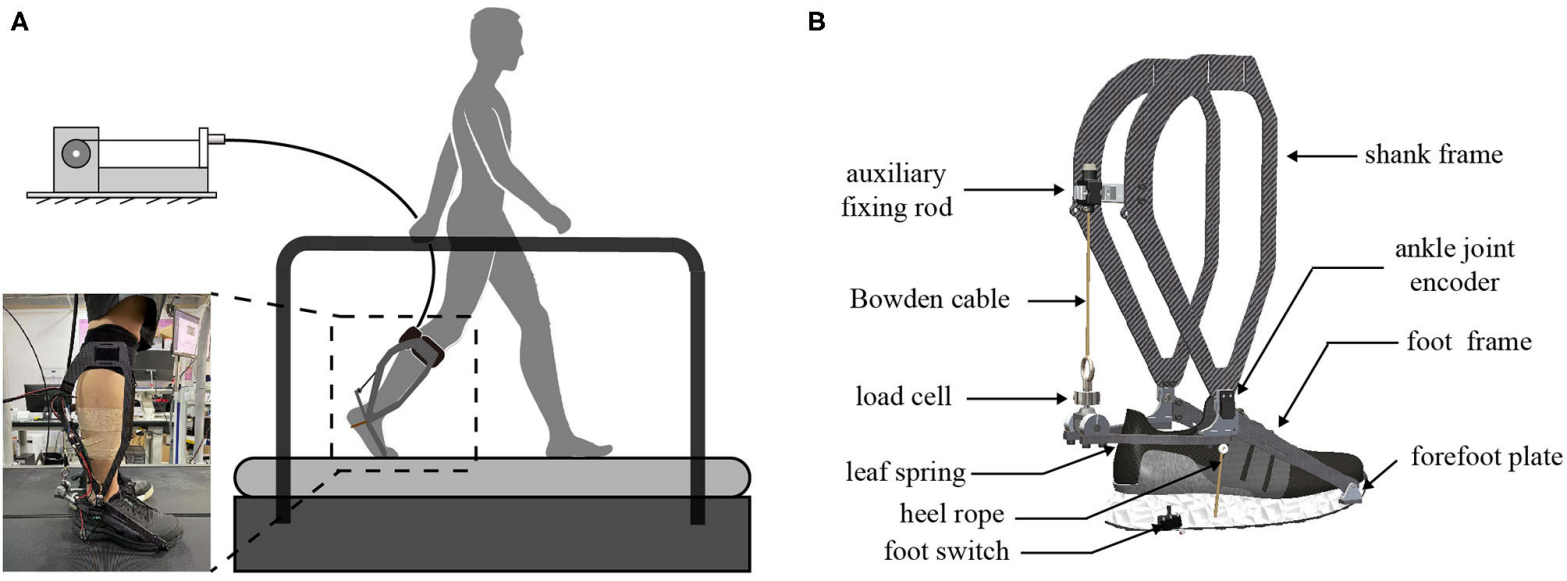
**(A)** Schematic of the tethered ankle exoskeleton system for human walking assistance. **(B)** The mechanical structure of the ankle exoskeleton.

The ankle exoskeleton system is comprised of an ankle exoskeleton, an off-board control system, a motor with its driver, and a uni-directional Bowden cable transmission that connected the motor with the exoskeleton end effector (Zhang et al., 2017b), as shown in Figure 1A. Using a tethered actuation system, the motor and driver can be mounted off-board, lowering the inertia of the device added to the wearer and decreasing the device interference to natural joint movements.

### 2.2. Ankle Exoskeleton Design

The ankle exoskeleton designed in this study was similar to a previously published version in Witte et al. (2015) but with some modifications to reduce the exoskeleton's weight and to improve wearing comfortability. It mainly consisted of three components: a shank frame, a foot frame, and a leaf spring as an ankle lever (Figure 1B). These three parts were joined together with two rotational shafts placed on each side of the human ankle. To reduce weight, these three components were all made of carbon fiber and were customized to fit different wearers' sizes. Considering that the exoskeleton was worn on the wearer's lower leg and directly interacted with the human body, the proximal of the shank frame was fixed with the wearer's calf by a Velcro strap to ensure comfortability. Meanwhile, the shank frame was hollowed out to avoid interfering sensor measurements and to reduce its weight. An aluminum alloy auxiliary fixing rod was used to connect the left and right shank frames. A flexible Bowden cable that passed through the rod connected the output shaft of the motor with the load cell which was fixed to a leaf spring. The customized leaf spring increased structure passive compliance, thus improving comfortability and safety of human-exoskeleton interaction (Zhong et al., 2021). The foot frame connected the exoskeleton joint shaft with the forefoot plate which was fixed under the exoskeleton shoe to ensure synchronous movement of the exoskeleton and human ankle. A lightweight synthetic rope was installed under the heel of the exoskeleton shoe to facilitate force transmission over the ankle joint.

A magnetic encoder (PQY18, Dongguan ACCNT Electronics Co., Ltd.) was installed on the lateral shaft of the exoskeleton to measure ankle joint angles. A load cell (DYMH-106, Bengbu DAYANG Sensor System Engineering Co., Ltd.) was used to measure Bowden cable tension. A footswitch (KW11-3Z-2, Shenzhen TELESKY Electronics Co., Ltd.) was installed on the outside of the heel of the exoskeleton shoe to detect heel strike. The total weight of one ankle exoskeleton was 577 g (534 g for the exoskeleton structure, 43 g for the sensors).

The actuation and control system were mounted on a shelf next to the treadmill. We actuated the exoskeleton using an AC brushless servo motor, a motor driver, and a 5:1 planetary gear (BSM90N-175AA, GBSM90-MRP120-5, and MF180-04AN-16A, ABB, Zurich, Switzerland). The AC brushless servo motor has low inertial and high power and is suitable for high speed and high torque applications. The motor driver was configured to velocity control mode. A digital optical encoder (E5, US Digital, WA, USA) was installed on the motor shaft to measure motor position. A high-speed real-time controller (DS1202, dSPACE, Paderborn, GmbH) was responsible for sampling sensory data with a frequency of 5 kHz and generating control commands with a frequency of 500 Hz.

### 2.3. Assistive Torque Profile

**Figure 2:**
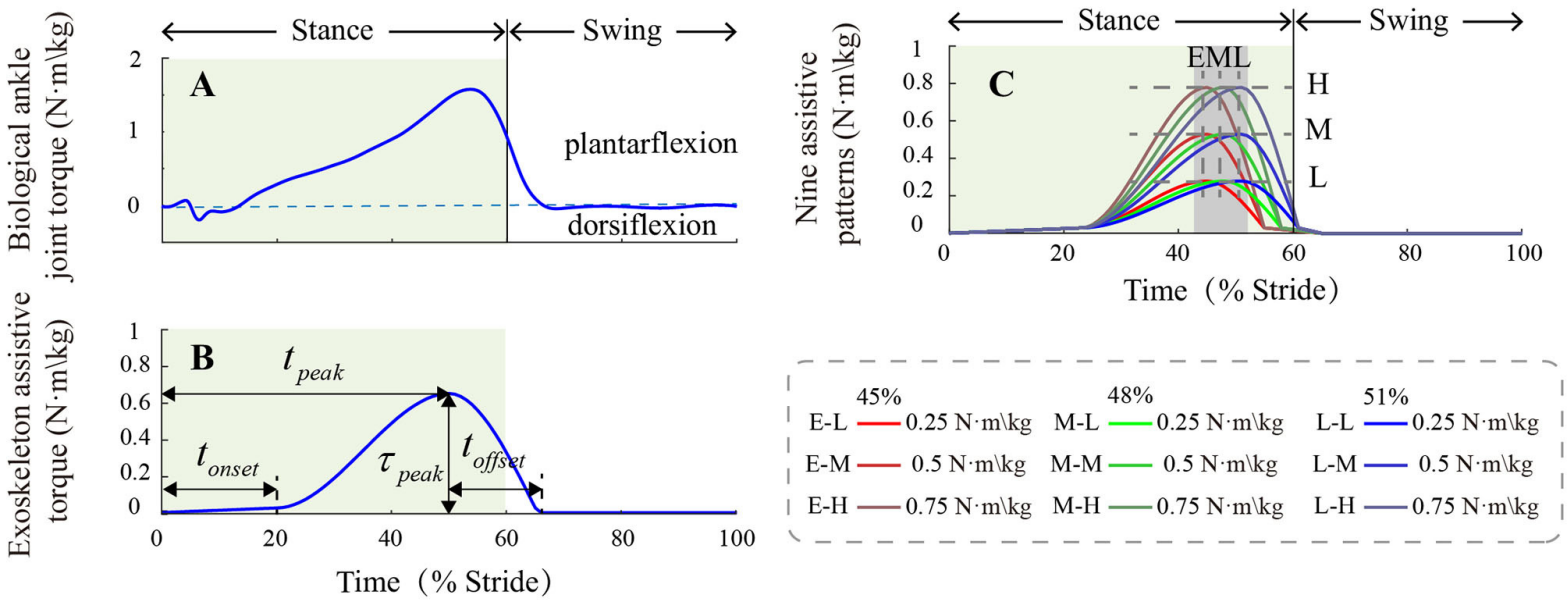
**(A)** Biological ankle moment during human level walking. **(B)** The ankle exoskeleton plantarflexion assistive torque profile and four assistance parameters: onset timing, peak torque timing, peak torque magnitude, and offset timing. **(C)** Nine assistance patterns that applied in the experiment. The light green rectangle shows the stance phase in a gait cycle.

Desired exoskeleton assistive torque profile was produced by emulating the biological ankle joint moment during human level walking (Figure 2). This single peak torque curve has been proved to be effective in previous studies (Jackson and Collins, 2015; Zhang et al., 2015). We used two cubic splines joined at their peaks to realize a continuous and smooth torque curve. Then, we defined four parameters from the curve: onset timing, peak torque timing, peak torque magnitude, and offset timing (Zhang et al., 2017b). Onset timing *t*_*on*_ represented the time of assistive torque onset. Peak torque timing *t*_*peak*_ represented the time that the exoskeleton provided maximum plantarflexion assistive torque. Peak torque magnitude τ_*peak*_ represented the maximum assistive torque that the exoskeleton provided for the wearer. Offset timing *t*_*off*_ represented the time from peak torque to zero. Onset timing, peak torque timing, and offset timing were normalized to percent stride and denoted by X%. Peak torque magnitude was normalized to body weight and denoted by *N*·*m*·*kg*^−1^.

We varied peak torque magnitude and peak torque timing independently within the parameter space of HILO and designed nine representative exoskeleton assistances. We set three different peak torque magnitudes in the experiment: 0.25 *N*·*m*·*kg*^−1^, 0.5 *N*·*m*·*kg*^−1^, and 0.75 *N*·*m*·*kg*^−1^ (referred to as Low, Medium, and High peak torque magnitudes respectively). We set three different peak torque time values in the experiment: 45, 48, and 51% of a gait cycle (referred as Early, Middle, and Late peak torque timings, respectively). These peak torque magnitudes and timing values could derive nine different assistance patterns (Figure 2). Compared to peak torque timing and magnitude, onset timing and offset timing had less effects on human walking biomechanics and energetics (Song and Collins, 2021). According to pilot tests, onset timing and offset timing were fixed to 23 and 10% of a gait cycle to ensure comfortability during the experiment.

### 2.4. Torque Control

**Figure 3:**
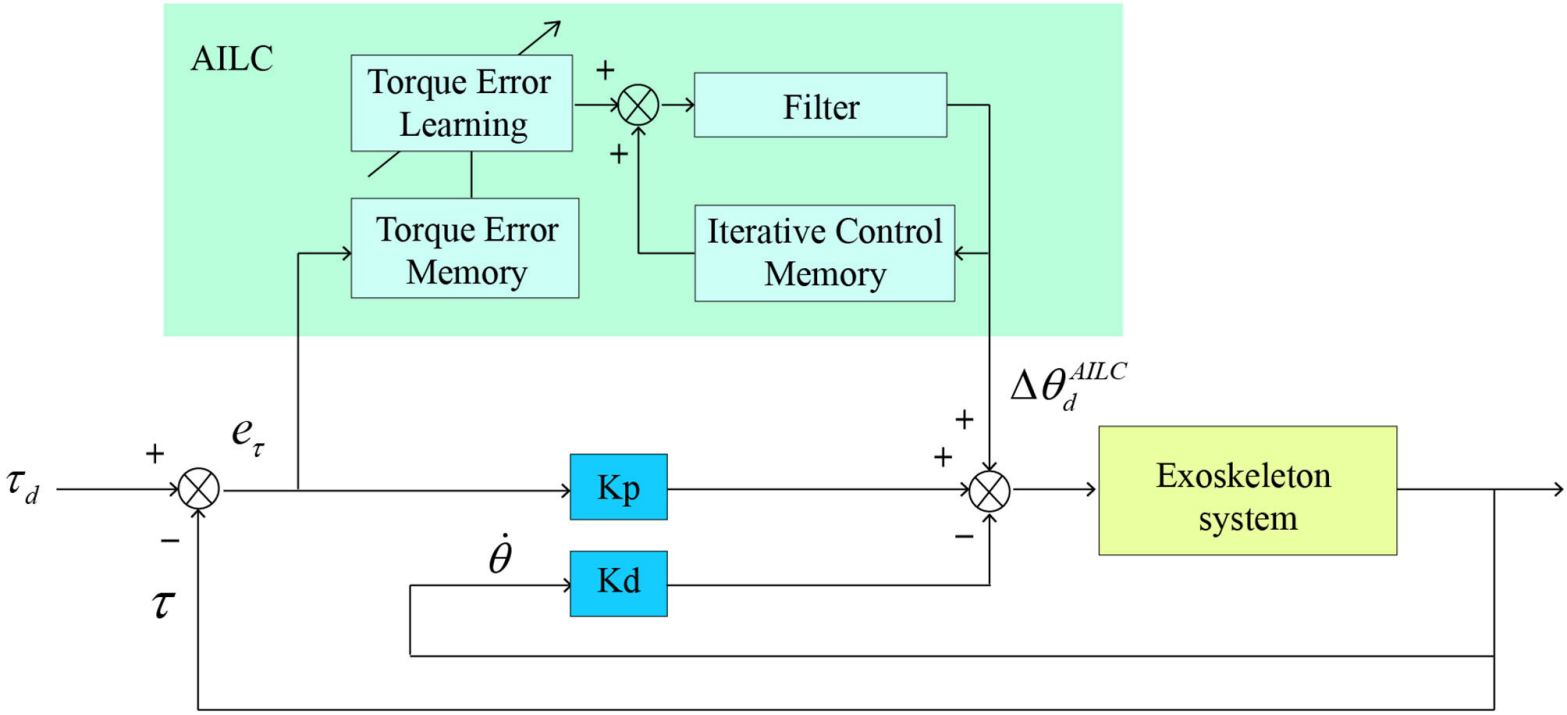
Schematic of the proportional control, damping injection, and adaptive iterative learning algorithm (AILC).

We implemented a torque controller that combined proportional control, damping injection, and adaptive iterative learning algorithm to improve torque tracking performance (Figure 3). Adaptive iterative learning algorithm (AILC) is suitable for nonlinear, periodical, and time-varying dynamic systems (Zhang et al., 2017a; Zhang and Collins, 2021). Desired motor velocity was computed as:


(1) $$\begin{array}{lll} {\overset{∙}{\theta }}_{d}\left(i,n\right) & = & \frac{1}{T}\cdot △\theta_{d}\left(i,n\right) \end{array}$$

(2) $$\begin{array}{lll} △\theta_{d}\left(i,n\right) & = & k_{p}\cdot e_{\tau }\left(i,n\right)-k_{d}\cdot \overset{∙}{\theta }\left(i,n\right)+△\theta_{d}^{AILC}\left(i+D,n\right) \end{array}$$

where ${\overset{∙}{\theta }}_{d}\left(i,n\right)$ is desired motor velocity, △θ_*d*_(*i, n*) is desired motor position increment, *e*_τ_(*i, n*) is torque tracking error, *T* is a parameter related to motor rise time, *k*_*p*_ and *k*_*d*_ are gains of proportional term and damping injection term, $\overset{∙}{\theta }\left(i,n\right)$ is measured motor velocity. $△\theta_{d}^{AILC}\left(i+D,n\right)$ is the learned feedforward compensation from the AILC. *i, n, D* represent time index, stride number, and the time delay between motor velocity command and motor rotation, respectively.

The AILC controller in Equation (2) was expressed as:


(3) $$\begin{array}{lll} △\theta_{d}^{AILC}\left(i,n+1\right) & = & \beta \cdot △\theta_{d}^{AILC}\left(i,n\right)-\rho \cdot k_{L}\cdot e_{\tau }\left(i,n\right) \end{array}$$

(4) $$\begin{array}{lll} \rho & = & \frac{\left|e_{\tau }\left(i,n\right)\right|}{\gamma } \end{array}$$

where β is a weight of the filter, *k*_*L*_ is the iterative learning gain, ρ is a torque error learning weight, γ is a parameter that is related to maximum torque tracking error.

**Figure 4:**
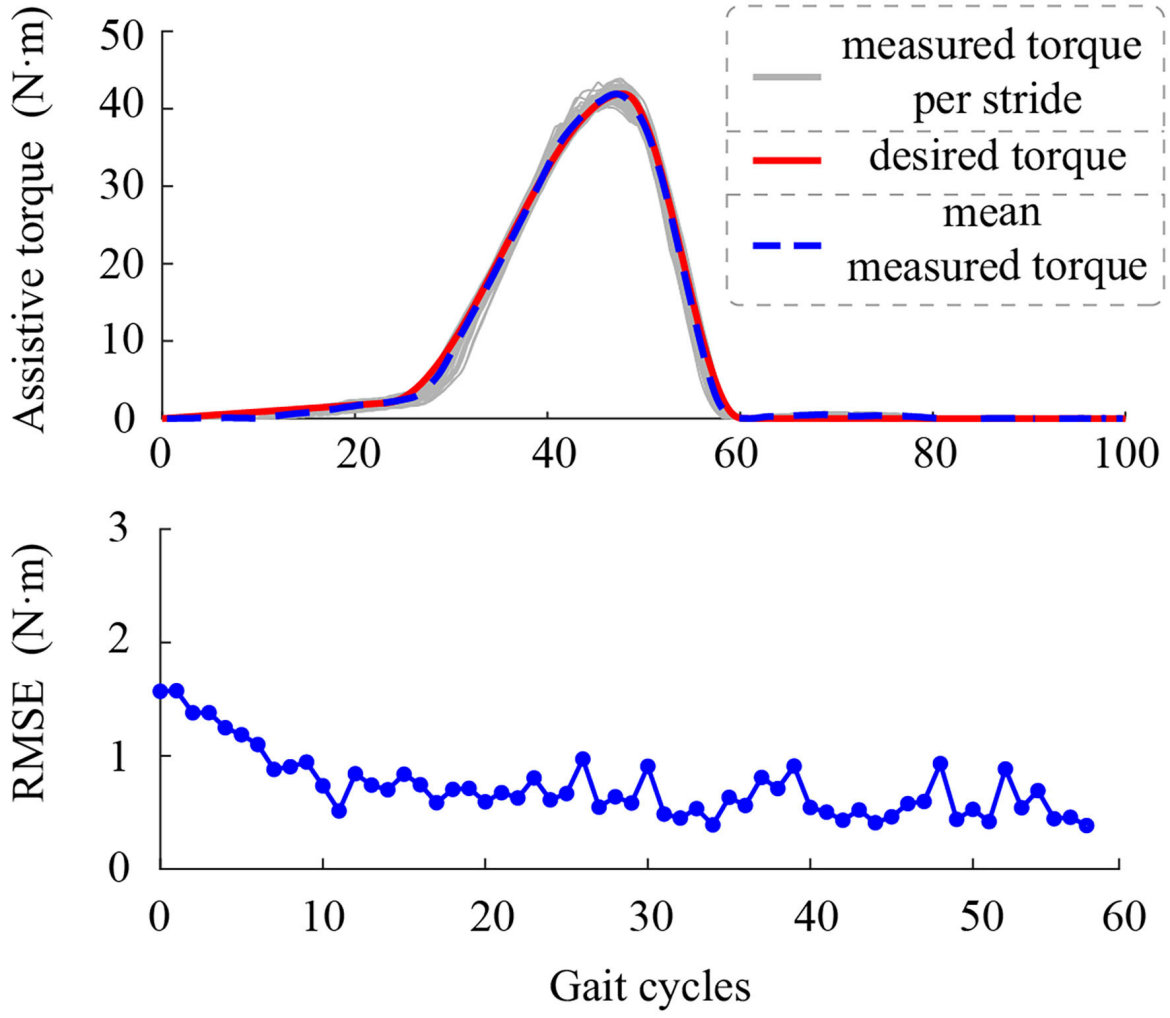
Torque tracking performance and root-mean-square error of each gait of the controller.

Controller parameters were systematically adjusted to minimize the tracking error (Table 1). We tested the performance of the proposed controller on one healthy subject that walked with the exoskeleton which the peak torque magnitude was set to 42 *N*·*m* (0.6 *N*·*m*·*kg*^−1^). Results show that the torque tracking root-mean-square-error is lower than 1 *N*·*m* within 10 gait cycles which means the proposed method could track desired torque accurately (Figure 4).

**Table 1:** Parameters values of the PD-AILC torque controller.

***k*_*P*_**	***k*_*D*_**	***k*_*L*_**	**β**	**γ**	**T**	**D**
8	0.01	0.2	1	2	0.050s	0.018s

### 2.5. Experimental Protocol

**Figure 5:**
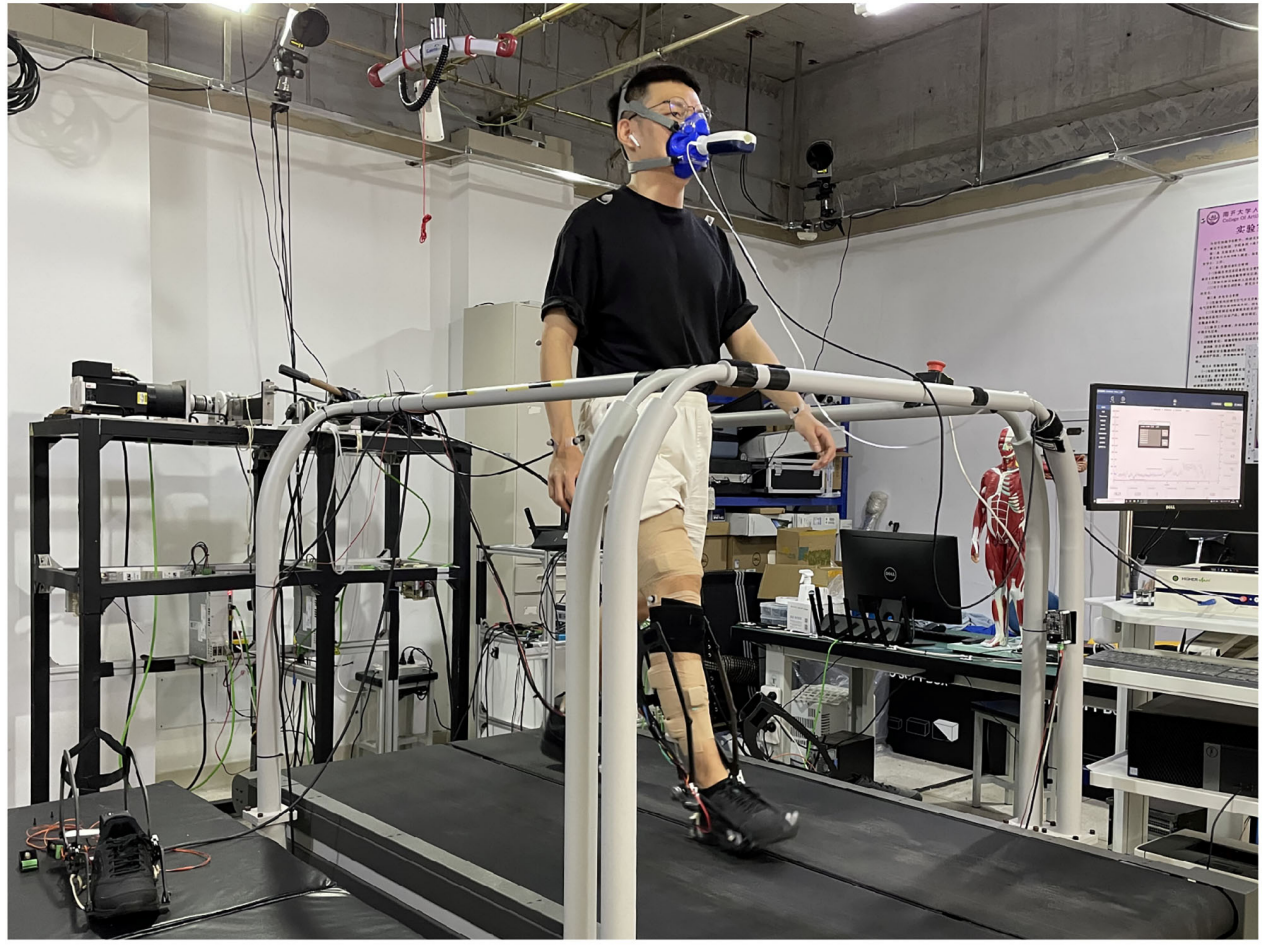
Photograph of human walking with an ankle exoskeleton.

We recruited 3 healthy subjects (age 24 ± 1.0 years; weight 65.3 ± 7.0 kg; height 1.75 ± 0.04 m; mean ± SD) to participate in the experiment. All experiments were conducted in a laboratory and performed by the participants walking on an instrument treadmill (Bertec, Columbus, OH, USA) at a fixed speed of 1.25 *m*·*s*^−1^ while wearing the ankle exoskeleton on the right ankle joint (Figure 5). The experiment was approved by the ethical committee of Nankai University. All participants were provided with written informed consent and volunteered to participate in the protocol.

**Figure 6:**
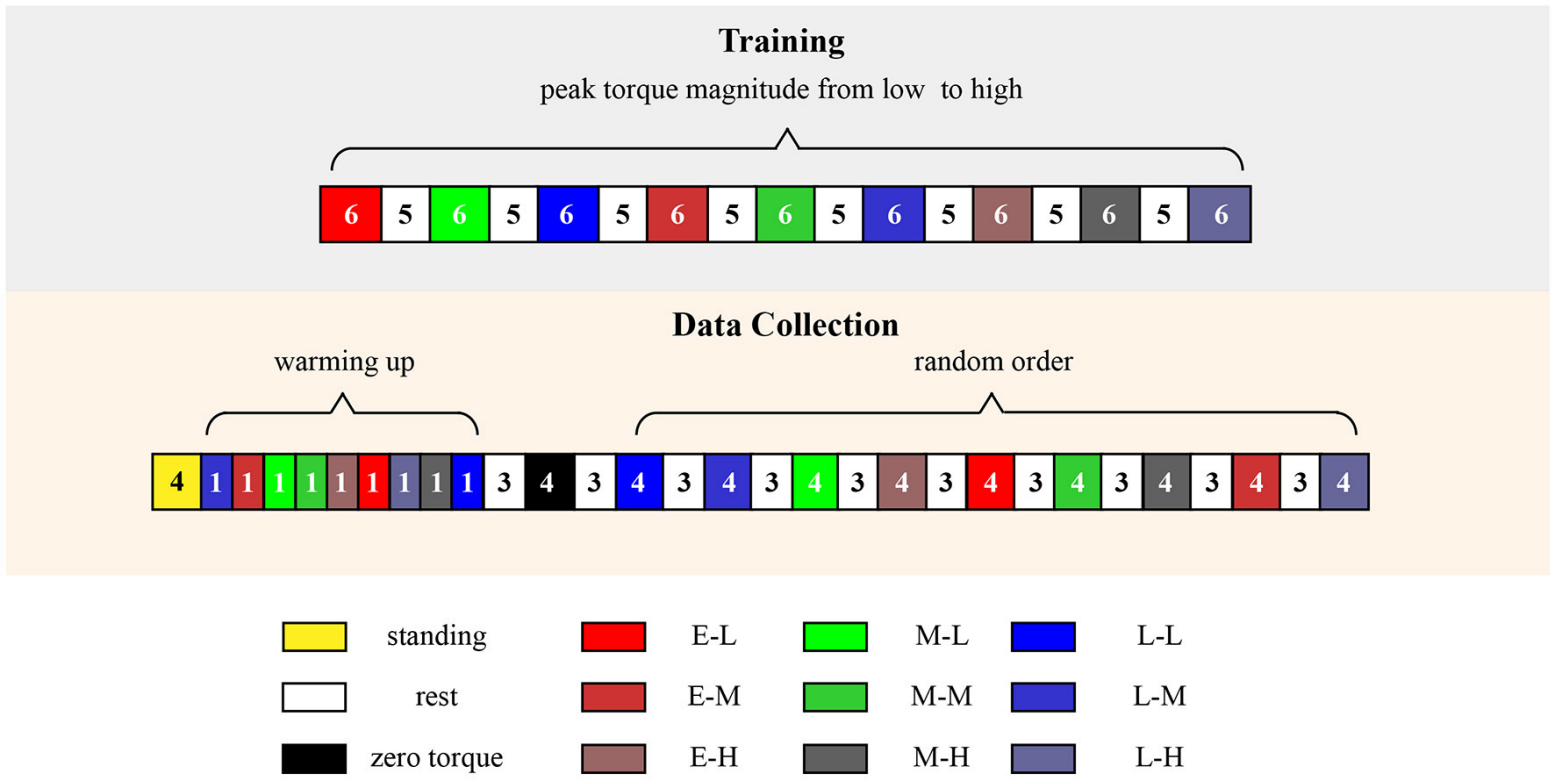
Experimental protocol which includes a training session and a data collection session. Numbers in the box represent the time of the corresponding condition.

The experimental protocol was split into a training session and a data collection session, each was done on a different day (Figure 6). In the training session, participants were trained to familiarize with the device and to walk with all assistance patterns in the order of low to high peak torque magnitudes. Each condition lasted 6 min and was followed by a 5-min break. During the training process, participants were advise to relax ankle muscles and to not resist the device.

Data collection was performed at least 24 h after the training session to avoid muscle fatigue. In the beginning, participants stood on the treadmill for 4 min to collect data on resting metabolic rate. Then, participants walked with all assistance patterns to warm up (1 min for each assistance pattern) and then took a 3 min rest. Subsequently, participants experienced ten 4-minute walking bouts: the zero torque assistance (ZT) in which the exoskeleton applied ZT to the human ankle and 9 powered assistance with different peak torque magnitudes and timing values. The 9 assistance patterns were applied randomly to minimize adaptation and learning effects. Each walking bout lasted 4 min to reach a steady-state metabolic rate. Resting breaks with an average of 3 min were given in between each walking bout to allow physical recovery.

### 2.6. Data Collection and Analysis

#### 2.6.1. Metabolic Cost

We measured *O*_2_ consumption and *CO*_2_ exhalation of the participants in each walking bout by using a portable pulmonary gas exchange measurement instrument (Smax58ce-sp, Nanjing Highermed Healthcare Technology Co., Ltd.). Then instantaneous whole-body metabolic rate was calculated by using the standard equation (Brockway, 1987):


(5) $$\begin{array}{l} E=0.278\cdot \overset{∙}{V}O_{2}+0.075\cdot \overset{∙}{V}CO_{2} \end{array}$$

where *E* is instantaneous metabolic cost, $\overset{∙}{V}O_{2}$ and $\overset{∙}{V}CO_{2}$ are measured flow rates. The steady-state metabolic cost was estimated by averaging the data of recorded metabolic rate during the last 2 min of each walking bout (Selinger and Donelan, 2014). Net metabolic cost for each walking bout was obtained by subtracting the standing metabolic rate from the calculated steady-state metabolic cost and then was normalized by the wearer's body weight. In each walking bout, participants were kept in moderate exercise and their respiratory exchange ratios were less than one.

A two-dimensional quadratic function was established to describe the relationship of peak torque timing (*t*_*peak*_), peak torque magnitude (τ_*peak*_), and averaged net metabolic cost (*E*). Coefficients of determination (*R*^2^) were calculated to evaluate how well the surface fits with the metabolic costs of different assistance patterns.

#### 2.6.2. Surface Electromyography

We measured sEMG signals of exoskeleton-side lower limb muscles *via* an invasive wireless EMG system (Trigno, Delsys, MA, USA) with a frequency of 5,000 Hz. Eight lower limb muscles were measured: SOL, tibialis anterior (TA), medial gastrocnemius (m.GAS), lateral gastrocnemius (l.GAS), rectus femoris (RF), vastus medialis (m.VAS), vastus lateralis (l.VAS), and semitendinosus (SEM). The skin was processed by a standard procedure (Rau, 2000) to reduce the impedance between the electrode and the skin. Medical tapes and elastic bands were used to wrap the electrodes to prevent them from falling during the experiment.

Raw EMG signals were preprocessed to extract the average linear envelope during each gait period. Raw EMG signals were high-pass filtered (second-order Butterworth, 20 Hz cut-off frequency), full-wave rectified, and low-pass filtered (second-order Butterworth, 10 Hz cut-off frequency). Subsequently, preprocessed EMG linear envelope amplitude was normalized by the average of the corresponding EMG peak value of the ZT condition for each participant. Muscle activity was quantified by calculating the root mean square (RMS) of normalized EMG envelopes from the last minute of each walking test.

#### 2.6.3. Ankle Joint Kinematics and Kinetics

Kinematics data was collected through a 10-camera optical motion capture system (Oqus 700+, Qualisys, Gothenburg, Sweden) at a sampling rate of 100 Hz. All participants worn 27 reflective markers during data collection (16 worn on the lower limb, 8 worn on the arm, 3 worn on the trunk). Exoskeleton-side ankle joint angle in the sagittal plane was calculated from measured marker data by using OpenSim 4.1 (Seth et al., 2018).

Ground reaction force (GRF) and center of pressure (COP) of the exoskeleton-side foot were collected by the instrument treadmill at a frequency of 5,000 Hz. Raw GRF and COP data were low-pass filtered with a second-order Butterworth filter with a 12 Hz cut-off frequency. Then we calculated ankle joint torque through inverse dynamic analysis in OpenSim 4.1 using processed GRF and COP data combined with joint angle. Joint torque calculated from inverse dynamics was a combination of biological ankle moment and exoskeleton assistive torque. Biological ankle moment was computed by subtracting the exoskeleton torque from calculated joint kinetics. Ankle joint angles and torques were calculated from the last 30 s of each walking condition.

### 2.7. Statistical Analysis

Paired *t*-test was conducted to compare the different powered conditions with the ZT condition to identify differences in metabolic cost, muscle activity, ankle joint kinetics, and kinematics. The significance level was set at α = 0.05. Statistical analyses were conducted with MATLAB (MathWorks, Natick, MA, USA).

## 3. Results

**Figure 7:**
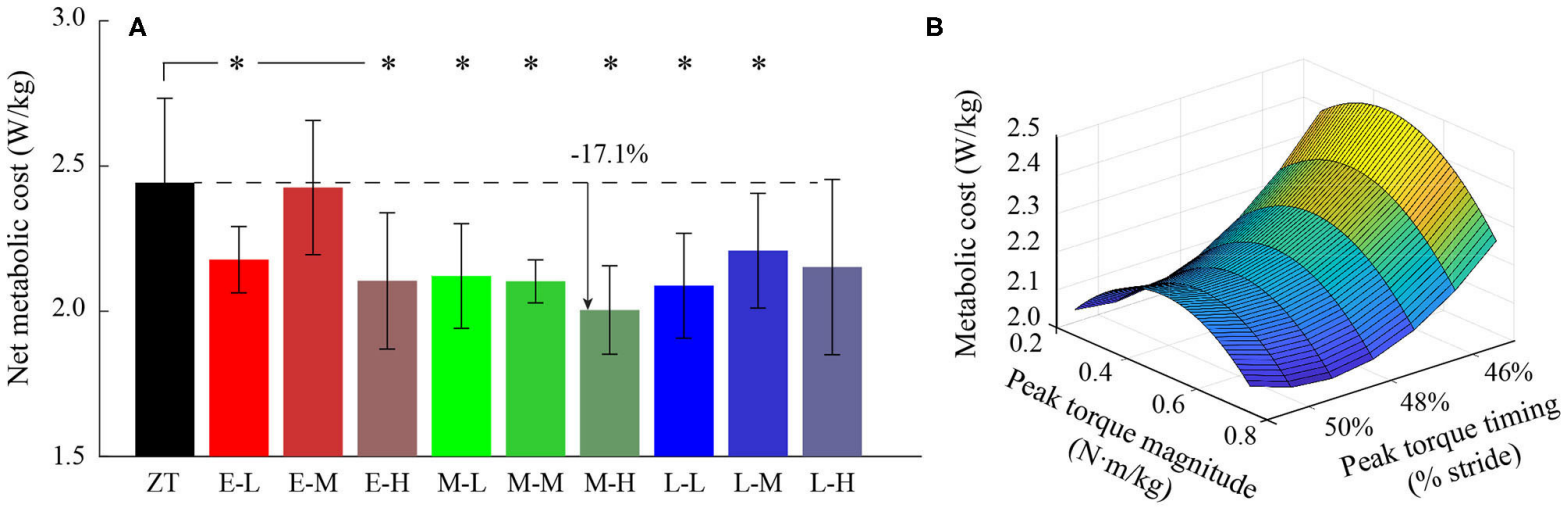
**(A)** Net metabolic cost in the ZT condition and nine assistance conditions. **(B)** Two-dimensional regression result for the relationship between peak torque timing, peak torque magnitude, and net metabolic cost. *represents statistically significant differences with respect to the ZT condition.

As shown in Figure 7A, the net metabolic cost was decreased during exoskeleton assisted walking compared to the ZT condition. Under the M-H condition, the metabolic cost was reduced by 0.44 ± 0.30 *W*·*kg*^−1^ relative to the ZT condition, which is a reduction of 17.1 ± 7.6% (P = 1.5·10^−11^). The average net metabolic reductions for the early, middle, and late peak torque timing conditions were 7.7 ± 8.8%, 14.1 ± 7.3%, and 11.6 ± 6.1%, respectively. The average net metabolic reductions for the low, medium, and high peak torque magnitude conditions were 12.1 ± 6.2%, 7.3 ± 8.5%, and 14.0 ± 7.7%, respectively. Changes in net metabolic cost for each condition can be found in Table 2. The second-order regression analysis showed that the relationship of net metabolic cost, peak torque timing, and peak torque magnitude which was expressed as:


(6) $$\begin{array}{l} E=35.52-134.4\;\cdot \;t_{peak}-0.14\;\cdot \;\tau_{peak}+133.3\;\cdot \;t_{peak}^{2}\\ \;+4.667\;\cdot \;t_{peak}\cdot \;\tau_{peak}-2.24\;\cdot \;\tau_{peak}^{2} \end{array}$$

**Table 2:** Energetics and biomechanics results during walking with different ankle exoskeleton assistances.

**Assistive pattern**	**Net Metabolic cost (*W*·*kg*^−1^)**	**Change in metabiloc cost (%)**	**Peak plantarflexion angle (**°**)**	**Peak dorsiflexion angle (**°**)**	**Peak biological angle torque (*N*·*m*·*kg*^−1^)**
Zero Torque	2.44 ± 0.29	-	21.5 ± 4.1	13.3 ± 4.3	1.27 ± 0.15
Early-Low	2.18 ± 0.11^*^	-9.9 ± 7.8%	21.9 ± 5.9	10.5 ± 4.3^*^	1.06 ± 0.20^*^
Early-Medium	2.43 ± 0.23	-0.3 ± 3.9%	21.7 ± 6.4	9.4 ± 6.3^*^	0.88 ± 0.21^*^
Early-High	2.10 ± 0.23^*^	-12.9 ± 10.2%	20.9 ± 4.3^*^	6.9 ± 6.1^*^	0.72 ± 0.21^*^
Middle-Low	2.12 ± 0.18^*^	-12.8 ± 3.3%	20.8 ± 4.7^*^	11.5 ± 5.4^*^	1.07 ± 0.16^*^
Middle-Medium	2.10 ± 0.07^*^	-12.6 ± 11.0%	22.3 ± 4.4	10.9 ± 7.1^*^	0.88 ± 0.17^*^
Middle-High	2.00 ± 0.15^*^	-17.1 ± 7.6%	25.6 ± 3.8^*^	8.1 ± 7.1^*^	0.68 ± 0.22^*^
Late-Low	2.09 ± 0.18^*^	-13.7 ± 7.7%	24.0 ± 2.7^*^	12.3 ± 4.6^*^	1.11 ± 0.14^*^
Late-Medium	2.21 ± 0.20^*^	-9.0 ± 5.5%	24.1 ± 1.7^*^	11.7 ± 6.0^*^	0.88 ± 0.15^*^
Late-High	2.15 ± 0.30	-12.0 ± 3.6%	24.2 ± 2.2^*^	8.5 ± 6.6^*^	0.60 ± 0.07^*^

* *Statistically significant differences with respect to the zero torque condition*.

This quadratic relationship (*R*^2^ = 0.76) can provide a prediction of the initial assistance pattern in HILO, which suggested metabolic cost is minimized under the condition of 49% peak torque timing and 0.75 *N*·*m*·*kg*^−1^ peak torque magnitude torque timing and 0.75 N·m·kg^−1^ peak torque magnitude (Figure 7B).

**Figure 8:**
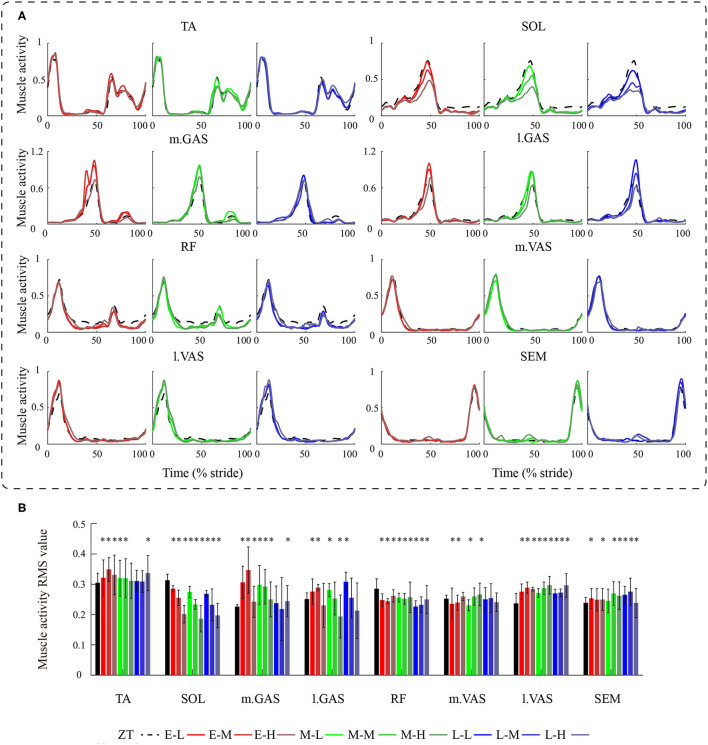
**(A)** Exoskeleton-side lower limb muscle activities during human walking with different ankle exoskeleton assistance patterns. **(B)** Muscle activity RMS values in different walking conditions. *represents statistically significant differences with respect to the ZT condition.

Muscle activity results are shown in Figure 8A and corresponding RMS values are shown in Figure 8B. The SOL muscle activity showed a large reduction in the late stance phase in all powered conditions. Under the M-H condition, the SOL activity RMS value was 0.186 ± 0.043, which is a reduction of 40.9 ± 19.8% compared to the ZT condition (P = 1.3·10^−10^). With increasing peak torque magnitude, the peak SOL activity decreased. The average muscle activity reductions for the low, medium, and high torque conditions were 11.6 ± 5.2%, 23.4 ± 8.8%, and 37.9 ± 9.5%, respectively. Similar trends were found in the l.GAS activity. The l.GAS activity decreased under the high torque conditions. Under the M-H condition, the l.GAS activity RMS value was reduced by 22.8 ± 29.2% relative to the ZT condition (P = 1.4·10^−6^). Other muscle activities showed no consistent changes compared to the ZT condition (Figure 8A).

**Figure 9:**
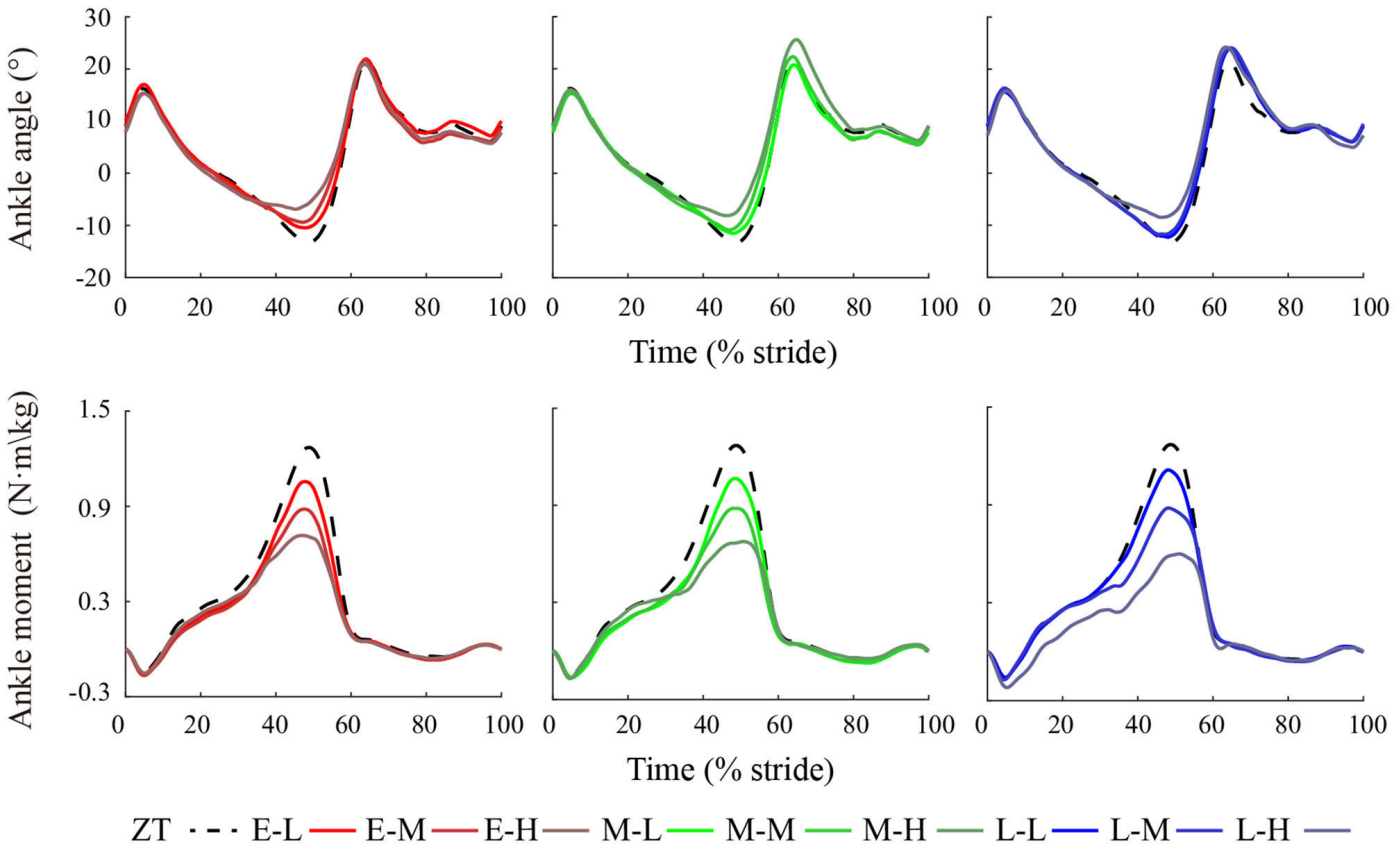
Exoskeleton-side ankle joint angle and biological ankle moment during human walking with different ankle exoskeleton assistance patterns.

As exoskeleton peak torque magnitude increased, maximum dorsiflexion angle was found to decrease and maximum plantarflexion angle was found to increase, as shown in Figure 9. Under the E-H condition, the maximum dorsiflexion angle was 6.9 ± 6.1°, which means 5.1 ± 1.4° lower than the ZT condition (P = 8.1·10^−19^). Under the M-H condition, the maximum plantarflexion angle was 25.6 ± 3.8°, which is a reduction of 4.6 ± 3.4° relative to the ZT condition (P = 4.5·10^−8^). Details of ankle kinematics are shown in Table 2.

Across the nine powered conditions, the biological ankle moment during ankle push-off decreased, as shown in Figure 9. Under the L-H condition, the average peak ankle moment was 0.60 ± 0.07 *N*·*m*·*kg*^−1^, which is a reduction of 0.58 ± 0.06 *N*·*m*·*kg*^−1^ compared to the ZT condition (P = 1.2·10^−10^). Compared to the low and medium peak torque magnitude assistance, the high torque assistance led to a large reduction of biological ankle moment. The average peak biological ankle moment reduction of the high torque conditions was 0.57 ± 0.09 *N*·*m*·*kg*^−1^. Peak biological ankle moment for each condition can be found in Table 2.

## 4. Discussion

This study investigated the effectiveness of a series of ankle exoskeleton assistance patterns on improving walking economy and provide a reference of initial pattern selection in HILO. In our experiments, participants walked on a treadmill while assisted by a lightweight cable-driven ankle exoskeleton. The assistance pattern was designed by emulating human ankle moment and was applied accurately to the wearers using proportional control, damping injection, and AILC. Nine assistance patterns that combined three peak torque magnitudes and three peak torque timing values were designed to represent the typical patterns within HILO parameter space.

Reduction in metabolic cost in all powered conditions indicated that the exoskeleton used in this study can effectively improve the walking economy for the wearers. While the magnitudes of metabolic cost reductions varied under different assistance patterns which suggested that the assistance pattern provided by the exoskeleton was able to influence the wearer's walking comfortability and adaptability. Under the M-H condition, the net metabolic cost was reduced most compared to other patterns which indicated that the M-H condition was effective in improving walking economy and can be selected as an initial pattern in HILO. A possible reason for the large metabolic cost reduction found in this condition may be due to the significant decreases in plantarflexor muscle activities and biological ankle moment. High peak torque magnitude can provide more power to the human ankle, while appropriate peak torque timing which is consistent with the instant of opposite leg heel contact during walking is also important to maximize power injection by the exoskeleton (Malcolm et al., 2015; Quinlivan et al., 2017).

A two-dimensional quadratic function was found to well characterize the relationship of metabolic cost, peak torque timing, and peak torque magnitude. When peak torque magnitude independently varied, the metabolic cost followed a convex pattern. An inverse trend was found when peak torque timing independently varied. Regression analysis indicated that the preferred peak torque timing is around 49% of a gait cycle, and the preferred peak torque magnitude is about 0.75 *N*·*m*·*kg*^−1^. This result is very close to a previous study which suggested that the average peak torque magnitude and timing of 11 subjects after optimization were 0.77 ± 0.08 *N*·*m*·*kg*^−1^ and 50.3 ± 1.4%, respectively (Zhang et al., 2017b). The small difference in peak torque timing may be caused by the position of the footswitch was in the lateral side of the exoskeleton shoe in this study, which differs from that placed inside the exoskeleton shoe, thus making the timing of heel contact detection late.

Calf muscle activities changed greatly than thigh muscles during exoskeleton assisted walking which was mainly because the assistance was applied to the ankle joint. Of the four measured calf muscles, SOL muscle activity showed the biggest reductions while walking with the exoskeleton. Moreover, SOL muscle activity decreased with increasing peak torque magnitude. But this trend was not observed in metabolic cost. It is difficult to attribute metabolic cost reduction only to the change in SOL activity. Based on walking biomechanics, with increasing torque magnitude, the SOL muscle contractions were more and more replaced by the exoskeleton assistance, resulting in more SOL muscle activity reduction. Similar trends were found in the l.GAS activity. The TA and m.GAS muscle activities increased under some conditions which may be caused by the co-contractions to counteract inappropriate patterns. The favorable assistance pattern may result from a trade-off between more SOL and l.GAS muscle activity reductions and less TA and m.GAS muscle activity increases. There were small variations observed in the RF, m.VAS, l.VAS, and SEM muscle activity. Variations of quadriceps femoris muscle activity may be due to the ankle exoskeleton assistance increased propulsion force during ankle push-off and indirectly assisted knee joint extension. Meanwhile, the impact to the ground of heel contact is increasing, resulting in higher muscle activity in the early stance phase.

Ankle joint angles were changed mainly during the mid to late stance phase in powered conditions and were changed little in other gait phases. This indicated that normal ankle functions were not interfered with by these patterns. The reduction of maximum dorsiflexion angle may imply that the Achilles tendon elongation was reduced due to energy absorption of the ankle exoskeleton. The stretch and recoil of the Achilles tendon can store and return elastic energy on the ankle joint, less elongation may imply that less energy is absorbed by the ankle joint and more energy is absorbed by the exoskeleton. The increases in maximum plantarflexion angles that were observed in the late timing conditions may be due to the exoskeleton assistance delayed ankle push-off, increased step length, thus increasing peak plantarflexion angle. We hypothesized that peak torque magnitude may influence more on dorsiflexion angle, while peak torque timing may influence more on plantarflexion angle.

The reduction of biological ankle moment in all powered conditions may indicate an effort to maintain the invariant total ankle moment of the wearer. The biological ankle moment was reduced more in the high peak torque magnitude patterns. Lower biological ankle moment reduced SOL muscle activity combined with smaller dorsiflexion angle suggested reduction of muscle-tendon force and Achilles tendon elongation. The assistance torque helped the recoil of the Achilles tendon to provide positive mechanical power during ankle push-off.

### 4.1. Conclusions

We investigated the effectiveness of different assistance patterns on improving walking economy prior to human-in-the-loop optimization on a lightweight cable-driven ankle exoskeleton. Results showed that the metabolic cost of walking was reduced by 17.1 ± 7.6% relative to the ZT condition under one assistance pattern, and SOL muscle activity was decreased by 40.9 ± 19.8% in that pattern. Besides, exoskeleton assistance changed maximum ankle dorsiflexion and plantarflexion angle and reduced biological ankle moment. Assistance pattern with 48% peak torque timing and 0.75 *N*·*m*·*kg*^−1^ peak torque magnitude was effective in improving walking economy and can be selected as an initial pattern in the optimization procedure. Data from our study provide a preliminary understanding of how humans interact with different external assistance patterns. Furthermore, the preferred assistance pattern in this study can be used as an initial value to improve the time efficiency of the human-in-the-loop optimization process.

## Data Availability Statement

The raw data supporting the conclusions of this article will be made available by the authors, without undue reservation.

## Ethics Statement

The studies involving human participants were reviewed and approved by Ethical Committee of Nankai University. The patients/participants provided their written informed consent to participate in this study. Written informed consent was obtained from the individual(s) for the publication of any potentially identifiable images or data included in this article.

## Author Contributions

JL and JZ: conceptualization and design of the experiments. WW and JC: ankle exoskeleton system development. WW and JD: performing experiments and analyzing data. WW: wrote the first draft of the manuscript. All the authors participated in the writing and revision of the manuscript.

## Funding

This study was supported in part by the Natural Science Foundation of China (62073179, 91848108).

## Conflict of Interest

The authors declare that the research was conducted in the absence of any commercial or financial relationships that could be construed as a potential conflict of interest.

## Publisher's Note

All claims expressed in this article are solely those of the authors and do not necessarily represent those of their affiliated organizations, or those of the publisher, the editors and the reviewers. Any product that may be evaluated in this article, or claim that may be made by its manufacturer, is not guaranteed or endorsed by the publisher.
